# Vitamin D on the susceptibility of gestational diabetes mellitus: a mini-review

**DOI:** 10.3389/fnut.2025.1514148

**Published:** 2025-02-03

**Authors:** Ting Zhang, Lan Yang, Shuman Yang, Song Gao

**Affiliations:** ^1^College of Physical Education and Health Sciences, Zhejiang Normal University, Jinhua, China; ^2^University Hospital, Zhejiang Normal University, Jinhua, China; ^3^School of Mathematical Sciences, Zhejiang Normal University, Jinhua, China

**Keywords:** vitamin D, gestational diabetes, insulin resistance, treatment, prevention

## Abstract

Gestational diabetes mellitus (GDM), which refers to diabetes mellitus or abnormal glucose tolerance of any degree occurring during pregnancy, is a distinct type within the diabetes classification. 25-hydroxyvitamin D deficiency has been associated with an increased risk of maternal glycaemia, insulin resistance and gestational diabetes. There is no consensus on the definition of vitamin D deficiency, but most scientists define vitamin D deficiency as less than 20 ng/mL (50 nmoL/L) of 25-hydroxyvitamin D. Vitamin D deficiency is common in women during pregnancy. Vitamin D can regulate the course of gestational diabetes, which may be related to regulation of insulin gene transcription, insulin secretion, intracellular and cytosolic calcium balance, inhibition of oxidative stress and inflammatory responses and alteration of glucose metabolism. This is a review article that aims to analyze the possible mechanism of vitamin D regulation of GDM, which provides a theoretical basis for clinical researchers in the future management of patients with GDM.

## Introduction

Gestational diabetes mellitus (GDM), a separate type of diabetes classification, is defined as diabetes or any degree of abnormal glucose tolerance that occurs during pregnancy and usually resolves postpartum (1). GDM is associated with an increased risk of adverse pregnancy outcomes such as hyperhydramnios, premature rupture of membranes, and fetal malformations (2). Newborns born of mothers with GDM are at increased risk of hypoglycaemia, hyperbilirubinaemia, and respiratory distress syndrome (3). One review showed that 21.4 million fetuses worldwide are affected by maternal hyperglycaemia during pregnancy each year (4). According to the International Diabetes Federation (IDF) in 2013, the global incidence of hyperglycemia during pregnancy in 20–49 year olds was 16.9%, of which the incidence of GDM was 14.2%. A survey showed that the prevalence of GDM in Chinese women aged 20–49 years was 8.6% (5). GDM is promoted by insulin resistance, release of insulin-antagonistic hormones and systemic inflammatory response during pregnancy (6). Compared with normal pregnant women, women with GDM have impaired pancreatic islet β-cell function and reduced adaptability to maintain normal blood glucose levels (7). Risk factors for GDM include advanced gestational age, overweight and obesity, family history of type 2 diabetes, and a history of GDM (8).

The most commonly used medications for GDM include metformin, probiotics and vitamin D (9). However, no interventions have been identified to prevent GDM. Insulin is the first-line treatment for gestational diabetes, but metformin and glibenclamide are not (10). Although there is evidence that metformin is safe and effective in treating GDM, its long-term effects are unknown. In addition, glibenclamide should be used with caution because it increases the risk of neonatal hypoglycaemia and macrosomia (11). Oral agents can be a safe and effective treatment for women with GDM. However, consideration should be given to the superimposed effect of comprehensive medication, such as whether insulin treatment for GDM and vitamin D supplementation will reduce or cause harm.

Serum vitamin D levels have been shown to be significantly higher in normal pregnant women than in those with GDM (12). Vitamin D deficiency during pregnancy may affect insulin secretion and increase insulin resistance, leading to an increased risk of GDM (13). In the latest meta-analysis, vitamin D deficiency and insufficiency are associated with miscarriage (14). There is evidence that vitamin D levels before pregnancy or in the first three months of pregnancy can be used as a biomarker to predict abortion (15). However, it has not been confirmed whether adjusting vitamin D levels can reduce the risk of miscarriage, and more well-designed randomized controlled trials (RCTs) are needed.

There is no consensus on the definition of vitamin D deficiency, but most researchers define vitamin D deficiency as a blood level of 25-hydroxyvitamin D (25-(OH)-D) below 20 ng/mL (50 nmoL/L). 25-hydroxyvitamin D levels of 30 ng/mL (75 nmoL/L) or higher are considered to be vitamin D sufficient (16). The recommended intakes should meet the requirements of the majority achieving a serum 25(OH)D concentration of at least 50 nmol/L considered by most committees as needed for maximum bone health for children and adults (17–19). The Institute of Medicine (IOM), in conjunction with Health Canada, recommend an intake of 600 IU/d for everyone from 1 to 70 years of age (17). For the same age group, the United Kingdom’s Scientific Advisory Committee on Nutrition recommended (SACN) 400 IU/d (18), World Health Organization (WHO) 200 IU/d, and European Food Safety Authority (EFA) 600 IU/d (19). In contrast, the Endocrine Society targeted 75 nmol/L to achieve maximum bone health (20).

There is a global trend toward increasing vitamin D deficiency in pregnant women (21). An Iranian study showed that 27% of 149 normal pregnant women had vitamin D levels below 25 nmol/L, and 73% had vitamin D levels between 25 and 74 nmol/L (22). A French cohort study showed widespread vitamin D deficiency in women in early pregnancy, with 50% of pregnant women having serum 25-(OH)-D levels below 20 ng/mL (23). A case–control study found serum 25-(OH)-D concentrations of 29.5 nmol/L in pregnant women and vitamin D deficiency in more than 90% of pregnant women (24). Another study found serum 25-(OH)-D concentrations of 22.7 ng/mL in rural Chinese women of childbearing age 18–44 years (25).

An Australian cohort study showed that women with serum 25-(OH)-D below 30 nmol/L in mid-pregnancy were more likely to develop GDM after adjustment for seasonal factors (26). A nested case–control study found a significantly higher prevalence of 25-(OH)-D deficiency or insufficiency in the GDM group, suggesting that vitamin D deficiency or insufficiency is a high risk factor for GDM (27). A prospective cohort study showed a significant positive association between serum 25-(OH)-D levels below 50 nmol/L in early pregnancy and the risk of developing GDM (OR = 2.82, 95% CI: 1.15–6.93) (28). A study by Vivanti et al. (29) found an increased risk of GDM in pregnant women with serum 25-(OH)-D less than 20 ng/mL in early pregnancy (OR = 1.42, *p* = 0.02), but no linear association was found. A linear analysis of one study showed a 2% reduction in the risk of GDM for each 10 nmol/L increase in serum 25-(OH)-D concentration (30). A U-shaped relationship was found between serum 25-(OH)-D concentration and the risk of GDM, i.e., pregnant women with serum 25-(OH)-D levels between 40 and 90 nmol/L had the lowest risk of GDM (31). In contrast, a case–control study by Azzam et al. (32) did not find a significant difference in vitamin D levels in patients with GDM compared to the control group. However, a significant correlation was found between vitamin D levels and glucose metabolism in patients with GDM, (HbA1c insulin, and HOMA-IR). The above observational studies suggest that vitamin D deficiency increases the risk of GDM. This review highlights the possible mechanisms by which vitamin D regulates GDM and provides a theoretical basis for clinical investigators managing patients with GDM.

## Search strategy

A comprehensive literature search was conducted in PubMed, EMBASE, and Web of Science using the following search terms: (vitamin D [Title] OR 25(OH)-D [Title] OR 25-hydroxy vitamin D [Title] OR vitamin D deficiency [Title]) AND (GDM [Title] OR gestational diabetes [Title] OR diabetic pregnancy [Title]) AND (randomized controlled trial [Title]). We excluded review articles and prioritized articles on RCTs with vitamin D supplementation as the main research method. Eleven articles were identified and used for data analysis. A summary of the included studies is presented in Table 1.

**Table 1:** Characteristics of the included studies.

References	Participants	Interventions	Outcomes
Gunasegaran et al. (63)	*n* = 70 Maternal age: 27.76 ± 3.93 y/25.66 ± 2.63 y Gestational age: 27–28 weeks	Vitamin D 1,000 IU and calcium 1,000 mg vs. vitamin D 250 IU and calcium 500 mg; 6 weeks	Vitamin D, FPG*, Insulin*, fasting lipid profile, GSH, LDL*, HDL*, total cholesterol*
Corcoy et al. (55)	*n* = 154 Maternal age: 32.2 ± 5.2 y/32.8 ± 5.4 y Gestation without GDM:≤19 + 6 weeks	1,600 IU/day vitamin D_3_ vs. placebo; 35–37 weeks	vitamin D*, FPG*, Insulin, HOMA-IR
Camarena Pulido et al. (54)	*n* = 54 women with GDM	Vitamin D 5,000 IU vs. placebo; 8 weeks	Vitamin D*, HbA_1c_, plasma glucose, Insulin, HOMA-IR, QUICKI
Jamilian et al. (71)	*n* = 60 Maternal age: 28.4 ± 6.2 y/29.6 ± 4.3 y Gestational age: 24–28 weeks	1,000 IU VD + 1,000 mg evening primrose oil vs. Placebo; 6 weeks	Vitamin D*, FPG*, Insulin*, HOMA-IR*, HOMA-B*, QUICKI*, TAG*, VLDL*, TC*, LDL*, HDL, TC/HDL*
Jamilian et al. (73)	*n* = 70 Maternal age: 18–40 y Gestational age: 24–28 weeks	50,000 IU VD every 2 weeks +1,000 mg omega-3 fatty acids twice a day vs. Placebo; 6 weeks	Vitamin D*, FPG*, Insulin*, HOMA-IR*, HOMA-B, QUICKI*, Triglycerides*, VLDL*, TC, LDL, HDL, TC/HDL
Jamilian et al. (70)	*n* = 58 Maternal age: 18–40 y Gestational age: 24–28 weeks	50,000 IU VD every 2 weeks + Probiotics 8 × 10^9^ CFU/day vs. Placebo; 6 weeks	Vitamin D*, FPG*, Insulin*, HOMA-IR*, QUICKI*, Triglycerides*, VLDL*, TC, LDL, HDL*, TC/HDL, Hs-CRP*, NO, TAC*, GSH, MDA
Jamilian et al. (74)	*n* = 60 Maternal age: 27.7–33 y Gestational age: 24–28 weeks	100 mg Mg + 4 mg Zn + 400 mg Ca + 200 IU VD twice a day vs. Placebo; 6 weeks	FPG*, Magnesium*, Zinc*, Calcium*, Vitamin D*, Hs-CRP*, Total nitrite, TAC*, GSH, MDA*
Karamali et al. (76)	*n* = 60 Maternal age: 18–40 y Gestational age: 24–28 weeks	100 mg Mg + 4 mg Zn +400 mg Ca + 200 IU VD twice a day vs. Placebo; 6 weeks	Magnesium*, Zinc*, Calcium*, Vitamin D, FPG*, Insulin*, HOMA-IR*, QUICKI*, Triglycerides*, VLDL*, TC, LDL, HDL, AIP*, AC*, CRR*
Asemi et al. (75)	*n* = 56 Maternal age: 18–40 y Gestational age:24–28 weeks	1,000 mg Ca/d plus VD 50,000 IU VD_3_ 2 times during the study (at baseline and at day 21 of the intervention) vs. Placebo; 6 weeks	Calcium, Vitamin D, FPG*, Insulin*, HOMA-IR*, HOMA-B, QUICKI*, TAG, LDL*, HDL*, Hs-CRP, NO, TAC, GSH*, MDA*
Mozaffari-Khosravi et al. (65)	*n* = 45 Maternal age: 30.7 ± 6.2 y/29.5 ± 4 y Gestational age: 24–28 weeks	300,000 IU VD_3_ vs. Placebo; 12 weeks	QUICKI*, β-cell function, Insulin sensitivity*, FPG, Glucose tolerance test, HbA_1c_, Calcium, HOMA-IR*, C-peptide*, Vitamin D*
Yazdchi et al. (69)	*n* = 72 Maternal age: 31.88 ± 4.0 y Gestational age: 24–28 weeks	50,000 IU of VD_3_/every 2 weeks vs. Placebo; 8 weeks	Vitamin D*, Glucose*, Insulin，HbA_1c_*, HOMA-IR, TC, Triglycerides, LDL, HDL, Hs-CRP*
Valizadeh et al. (68)	*n* = 96 Maternal age: 32 ± 5 y Gestational age: 12–32 weeks	700,000 IU VD_3_ vs. Placebo; 12 weeks	Vitamin D*, FPG, 2-hPLG, Serum Insulin, HOMA-IR, HbA_1C_

FPG, fasting plasma glucose; HOMA-IR, homeostasis model of assessment-estimated insulin resistance; HOMA-B, homeostasis model of assessment-estimated B cell function; QUICKI, quantitative insulin sensitivity check index; TAG, triacylglycerol; VLDL, very low-density lipoprotein; TC, total cholesterol; LDL, low-density lipoprotein; HDL, high-density lipoprotein; Hs-CRP, high sensitivity C-reactive protein; NO, nitric oxide; TAC, total antioxidant capacity; GSH, total glutathione; MDA, malondialdehyde; AIP, atherogenic index of plasma; AC, atherogenic coefficient; CRR, cardiac risk ratio; 2-hPLG, 2-h post 75 g glucose load plasma glucose. *indicates statistically significant differences between the intervention group and the control group (*p* < 0.05).

### Metabolism and physiological role of vitamin D

Vitamin D is a fat-soluble vitamin with calcitriol bioactivity. Vitamin D_2_ and vitamin D_3_ are the most abundant in the human body, with vitamin D_3_ accounting for 90–95% of total vitamin D in the body. Vitamin D_2_ only comes from plant sources (mushrooms), while vitamin D_3_ comes from animal sources, such as cod liver oil, egg yolk and liver (33). Vitamin D_3_ is mainly synthesized through the skin in response to UV radiation, which is the main way the body obtains vitamin D (34). Vitamin D is converted to 25-(OH)-D by secondary hydroxylation in the liver and kidneys, where it exerts its biological effects. Vitamin D is also a hormone promoter involved in biological processes such as calcium and phosphorus metabolism, immunomodulation, and anti-inflammation (35, 36). Vitamin D_2_ and vitamin D_3_ are catalyzed in the liver by D_3_-25-hydroxylase to form 25-(OH)-D_3_, which is secreted into the blood by the liver and transported to the kidney by vitamin D-binding proteins. Catalyzed by 25-(OH)-D_3_-1α-hydroxylase and 25-(OH)-D_3_-24-hydroxylase, 25-(OH)-D_3_ is further oxidized to 1,25-(OH)-D_3_ and 24,25-(OH)-D_3_. 1,25-(OH)_2_-D is the active form of vitamin D. It acts on target organs such as the small intestine, kidney, and bone and is involved in the regulation of calcium and phosphorus metabolism (37).

Vitamin D requirements are high at certain stages of life, such as embryonic development, infancy, early childhood, adolescence, and pregnancy (38). Studies have shown that the nutritional and environmental factors experienced during the critical period of prenatal life affect the growth of the fetus and the development of organs and system functions (39). At this programming stage, these persistent changes in various physiological processes can lead to changes in gene expression patterns and then affect phenotype and function through epigenetic mechanisms. To meet embryonic developmental and immune requirements, 1,25-(OH)_2_-D synthesis is increased in the kidney during pregnancy. The meconium and placenta produce large amounts of 1,25-(OH)_2_-D by increasing CYP27B1 hydroxylase activity (40). Serum 1,25-(OH)_2_-D levels can be twice as high in late pregnancy as in non-pregnant women (41). However, low outdoor light exposure, low dietary vitamin D content, altered body metabolism and obesity increase the rate of vitamin D deficiency (42). Low maternal serum 25-(OH)-D levels are associated with adverse pregnancy outcomes, such as pre-eclampsia, small for gestational age, and neonatal hypocalcemia (43). Studies have shown that maternal vitamin D deficiency may increase the risk of GDM and pre-eclampsia (44). Vitamin D supplementation during pregnancy may reduce adverse pregnancy outcomes such as cesarean section, hyperhydramnios, neonatal asphyxia, macrosomia and premature rupture of membranes (45).

Vitamin D is converted to 1,25(OH)_2_D_3_ *in vivo* by catalytic enzymes in the liver and kidneys (46, 47). When liver function declines, the hydroxylation of vitamin D in the liver is not affected, whereas when renal function declines, the further hydroxylation of 25-hydroxyvitamin D in the kidney may be affected, resulting in a decrease in the level of 1,25(OH)_2_D_3_, which leads to a decrease in intestinal calcium absorption (48). However, most of the 1,25(OH)_2_D in the maternal circulation is produced by the kidneys. A higher level of 1,25(OH)_2_D is essential to increase intestinal calcium absorption during pregnancy and to support calcium metabolism in the mother and fetus (49). In addition, 1,25(OH)_2_D is involved in regulating the immune function during pregnancy (50).

### Preventive effect of vitamin D supplementation on GDM

The rates of positive glucose provocation test (34.8% vs. 11.4%) and positive glucose tolerance test (35.6% vs. 10.9%) were significantly higher in pregnant women without vitamin D supplementation in early and mid-pregnancy than in pregnant women supplemented with 5,000 IU of vitamin D per day (38). A 10-year follow-up program showed a significantly lower risk of GDM in pregnant women who supplemented with either 1 to 399 IU or more than 400 IU of vitamin D per day compared with those who did not (RR values of 0.86 and 0.71, respectively) (51). The incidence of GDM was significantly lower in pregnant women who received 300,000 IU of vitamin D intramuscularly between 16 and 20 weeks gestation than in those who received 400 IU of vitamin D orally daily (52).

A study by Shahgheibi et al. (38) conducted a randomized controlled trial in 100 women with high risk factors for GDM in early pregnancy. Pregnant women in the intervention group received 5,000 IU of vitamin D daily up to 26 weeks of gestation, and the control group received placebo. The results showed a significantly lower incidence of GDM in the intervention group than in the control group (11.4% vs. 34.8%; *p* = 0.01). However, some other studies revealed contradictory results (53–55). One study (56) randomized 179 pregnant women with <20 weeks gestation and serum vitamin D levels below 30 μg/L into two groups. The intervention group was supplemented with 50,000 IU of vitamin D every 2 weeks, and the control group was supplemented with 400 IU of vitamin D daily until delivery. The results showed that the incidence of GDM in the two groups was 8 and 13%, respectively (*p* = 0.25), indicating that high-dose vitamin D supplementation starting at a mean of 14 weeks of gestation did not improve blood glucose levels during pregnancy. It is worth noting that neither of the above studies provided dietary advice in early pregnancy. Similar results were found in another study by Zhao et al. (57), i.e., although vitamin D supplementation in early pregnancy significantly improved maternal vitamin D levels, it did not significantly reduce the incidence of GDM.

Multivitamin supplements during pregnancy usually contain only 200–400 IU. This dose is sufficient for the general population, but is too low to treat vitamin D deficiency, especially in vitamin D deficient mothers and newborns (58). Due to the lack of evidence to determine the appropriate amount of vitamin D supplementation during pregnancy, the recommended level of vitamin D intake is quite conservative. There is no consensus on the optimal vitamin D status during pregnancy, that is, the serum 25(OH)D level is defined as 50 nmol/L-75 nmol/L (59). Institute of Medicine and the US Endocrine Society agree on limiting the dose to 4,000 IU/day or even higher, but only for a short time in the third trimester and always under the supervision of an obstetrician (20). However, excessive vitamin D intake during pregnancy is associated with a risk of fetal hypercalcaemia. Furthermore, too much vitamin D in the blood (more than 375 nmol/L or 150 ng/mL) can cause nausea, vomiting, muscle weakness, confusion, pain, loss of appetite, dehydration, excessive urination, thirst and kidney stones (60).

### Therapeutic effects of vitamin D supplementation on GDM

Vitamin D supplementation may improve glucose metabolism in women with GDM by reducing fasting glucose, glycated hemoglobin, and serum insulin levels (61). Although the relevance of vitamin D to GDM is currently controversial, vitamin D is considered a potential candidate for the treatment of GDM (62, 63). Studies have shown that supplementation with 50,000 IU of vitamin D every 2 weeks improves insulin resistance and reduces fasting insulin and total cholesterol levels (64).

A single injection of 300,000 IU vitamin D_3_ can maintain serum 25-hydroxyvitamin D at 50–80 nmol/L within 3 months, which can safely and effectively improve the vitamin status and insulin resistance index of pregnant diabetic mothers after delivery (65). A study by Asemi et al. (62) randomized 54 pregnant women with GDM to vitamin D supplementation or placebo in two groups. Pregnant women in the vitamin D supplementation group (*n* = 27) received 50,000 U vitamin D on the day of enrollment (day 0) and on day 21, and the placebo group (*n* = 27) received a placebo at the same time. After 6 weeks, a significant decrease in fasting glucose, serum insulin and insulin resistance levels, and a significant increase in insulin sensitivity were found in the intervention group without changing their daily physical activity or dietary intake. The incidence of excess amniotic fluid and neonatal hyperbilirubinemia was reduced with vitamin D supplementation in pregnant women with GDM compared with the placebo group. A study by Wang et al. (66), which included 19 randomized controlled studies, found that vitamin D supplementation significantly reduced fasting glucose and insulin concentrations, improved insulin resistance, and reduced the risk of adverse maternal and infant pregnancy outcomes in women with GDM. Another systematic review found that vitamin D supplementation in women with GDM may reduce fasting glucose levels and the risk of maternal and neonatal hospitalization and the neonatal complications (e.g., hyperbilirubinemia, amniotic fluid excess) (67). However, moderate-to-high quality evidence was not available due to the small number of included studies. A study by Valizadeh et al. (68) conducted a randomized controlled study in women diagnosed with GDM at 12–32 weeks. A total of 700,000 IU of vitamin D3 was administered to women in the intervention group and only serum 25-(OH)-D levels differed between the two groups, but no significant differences in fasting glucose, fasting insulin, glycosylated hemoglobin or insulin resistance were found between the two groups. A study by Yazdchi et al. (69) found that although vitamin D supplementation reduced fasting blood glucose levels, there was no significant improvement in insulin and insulin resistance levels.

In addition to vitamin D supplementation alone, combined supplementation with vitamin D and other nutrients is a potential option for the treatment of GDM. A study showed that vitamin D and probiotics supplementation for 6 weeks may improve glucose and lipid metabolism in women with GDM (70). Jamilian et al. (71) investigated the effects of vitamin D and EPO on insulin resistance and lipid concentrations in women with GDM. Sixty GDM patients were randomized to treatment and control groups. The experimental group received 1,000 IU vitamin D_3_ and 1,000 mg EPO, while the control group received placebo. After 6 weeks of intervention, fasting blood glucose (−3.6 ± 7.5 vs. +1.5 ± 11.4 mg/dL, *p* = 0.04), serum insulin concentration (−2.0 ± 5.3 vs. + 4.6 ± 10.7 μIU/ml, *p* = 0.004) and homeostasis model of assessment (HOMA) were observed in the experimental and control groups. However, the researchers did not observe the effect of vitamin D and EPO supplementation on serum HDL concentration. After taking 1,000 mg of *ω*-3 fatty acids daily for 6 weeks, GDM patients’ insulin resistance also improved, but their blood glucose, insulin sensitivity and blood lipids did not change (72). Another study showed that the combination of vitamin D and ω-3 fatty acids for 6 weeks had beneficial effects on fasting blood glucose, serum insulin levels, the steady-state model of insulin resistance, the quantitative index of insulin sensitivity, serum triglycerides and very low density lipoprotein cholesterol levels in patients with GDM (73). In a study by Jamilian et al., patients with GDM were randomized into three groups to receive vitamin D (50,000 IU/every 2 weeks) plus probiotics (8 × 10^9^ CFU/day) (n/30), probiotic (8 × 10^9^ CFU/day) (n/29), or placebo (n/28) for 6 weeks each (70). Vitamin D combined with probiotics significantly reduced fasting blood glucose (β-10.99 mg/dL; 95% CI, −14.26, −7.73; *p* < 0.001), serum insulin level (β-1.95 μIU/ml; 95% CI, −3.05, −0.84; *p* = 0.001) and steady-state assessment model insulin resistance (β-0. 76; 95% CI, −1.06, −0.45; *p* < 0.001). Therefore, the combination of vitamin D and probiotics has a beneficial effect on the metabolic status of women with GDM. A study by Jamilian et al. (74) in Iraq showed that a 6-week combined magnesium-zinc-calcium vitamin D supplementation intervention was effective in improving insulin sensitivity and reducing lipid levels, inflammation and biomarkers of oxidative stress in women with GDM.

A randomized controlled study included 56 pregnant women with GDM who were not on insulin therapy. The trial group took 100 mg of calcium and 50,000 IU of vitamin D daily at baseline and on day 21. The placebo group took a placebo at the same times. The results showed a significant reduction in fasting glucose, serum insulin levels and insulin resistance levels in the study group (75). In another Iranian study, 60 pregnant women with GDM who were not on insulin therapy were randomized to receive placebo or a combination of magnesium-zinc-calcium-vitamin D supplementation for 6 weeks. All participants maintained their daily diet and physical activity throughout the study. The results showed that combined magnesium-zinc-calcium-vitamin D supplementation significantly reduced fasting glucose, serum insulin, insulin resistance, and serum high-sensitivity C-reactive protein levels, and improved insulin sensitivity and total antioxidant capacity *in vivo* (76). A study by Karamali et al. (77) found a significant reduction in cesarean delivery rates in pregnant women with GDM who took combined calcium + vitamin D supplementation in the absence of other supplements compared with those who took placebo. No macrosomes were formed in the combined supplementation group, whereas the incidence of macrosomes in the placebo group was 13.3% (*p* = 0.03), suggesting that combined supplementation with vitamin D and other nutrients is beneficial for pregnant women with GDM.

### Mechanisms by which vitamin D reduces the risk of GDM

Vitamin D and its active metabolites play a role in insulin resistance, which is the basis for the development of GDM. Vitamin D has emerged as an important idea for the treatment of GDM (78), and it may modulate the development of GDM in the following ways (79).

- Vitamin D regulates insulin secretion by binding to the vitamin D receptor (VDR), which is widely distributed in various cells in the body, including islet β cells, islet α cells, PP and D cells, and is involved in regulating the physiological processes of glucose metabolism (80). Vitamin D can stimulate insulin receptor expression and enhance insulin-mediated glucose transport by binding to VDRs and vitamin D-dependent calcium-binding proteins in pancreatic β-cells to lower blood glucose levels (81).
- Vitamin D can maintain mitochondrial respiratory chain activity directly or through VDR in the nucleus. Vitamin D deficiency leads to decreased mitochondrial respiration, decreased ATP formation, increased reactive oxygen species formation, and decreased insulin signaling pathway activity (82).
- Vitamin D regulates insulin gene transcription. VDR binds specifically to 1,25-(OH)-D3 to form a heterodimer that binds to the vitamin D cis-response element on the DNA binding region and regulates insulin gene transcription and blood glucose levels (83).
- Vitamin D regulates intra-and extracellular calcium homeostasis. Vitamin D accelerates insulin secretion from pancreatic β-cells by regulating the intracellular flux of calcium ions and upregulating the transcriptional expression level of insulin mRNA. Vitamin D regulates calcium-dependent peptidase activity, which induces the conversion of proinsulin to insulin, and promotes insulin secretion by regulating pancreatic β-cell proliferation and apoptosis (84).
- Vitamin D inhibits the conversion of preadipocytes to mature adipocytes, thereby suppressing adipogenesis and reducing insulin resistance in peripheral tissues. The relationship between vitamin D and obesity is bidirectional (85). Vitamin D deficiency can aggravate obesity and related metabolic complications (86, 87). Conversely, obesity can aggravate vitamin D deficiency (88). In obesity, the metabolic disorder of vitamin D may be related to the imbalance of intestinal ecology and the decreased activation of vitamin D in liver and adipose tissue (83, 89).
- Vitamin D deficiency can exacerbate pregnancy-induced insulin resistance, which can lead to GDM.

In late pregnancy, maternal antagonistic insulin-like substances increase, insulin sensitivity decreases, and maternal glucose and free fatty acid concentrations increase (90). Insulin may be overproduced to maintain normal blood glucose levels. At this time, Ca^2+^ and reactive oxygen species signaling is excessive, islet β-cell function is impaired, and even islet cell death occurs, leading to GDM (91).

- Vitamin D can inhibit oxidative stress and the inflammatory response, which in turn prevents the onset of GDM. A variety of inflammatory mediators are produced by the placenta including during pregnancy. Vitamin D is a potential immunosuppressive agent that down-regulates pro-inflammatory markers such as TNF-α and IL-2 (92). The increase of serum concentration of 25-(OH)-D is related to the increase of CD 38^+^ expression on B cells and the decrease of T cell-dependent proinflammatory cytokines (93).
- In vitamin D-deficient pregnant women, increased pro-inflammatory cytokines lead to enhanced pro-inflammatory and oxidative stress responses and endothelial dysfunction (94). The antioxidant effect of vitamin D can cause apoptosis of reactive oxygen species clusters and eliminate direct reactive oxygen species cluster damage to β-cells (38).

In addition, vitamin D can play indirectly: (1) low vitamin D stimulates PTH secretion, while PTH promotes insulin resistance and obesity, by inhibiting lipolysis (95, 96); (2) by its interaction with other hormones, e.g., glucocorticoid, sex hormone and renin-angiotensin-aldosterone system (RAAS) (87, 97); (3) by its negative effects on lipolysis (98); (4) by its influence on the increased osteocalcin and adiponectin (99, 100).

## Conclusion

The prevention and management of GDM has become a global issue in maternal and child nutrition. A large number of clinical studies have supported the conclusion that women with vitamin D deficiency are at higher risk of GDM (101). However, whether vitamin D is directly involved in the pathogenesis of GDM and the pathophysiological mechanisms involved remain unclear. The mechanism between vitamin D and GDM is still under investigation and may act by improving insulin sensitivity, promoting insulin secretion, and inhibiting the development of islet β-cell apoptosis and inflammatory responses. Unfortunately, at the moment there is a lack of consensus on optimal intake and serum levels of vitamin D during pregnancy, which makes it difficult to establish general public health recommendations, which can differ between different populations or regions. Additionally, varying doses of vitamin D used in these trials make it difficult to make a clear conclusion. Additional multicenter randomized well-controlled trials need to be designed to investigate more deeply the relationship between vitamin D and GDM.
